# The Correlation Analysis for New Media Internet Celebrity Economy in College Students’ Entrepreneurial Values and Entrepreneurial Behavior

**DOI:** 10.3389/fpsyg.2022.892347

**Published:** 2022-06-16

**Authors:** Yonghui Xiang, Weiwei Wang

**Affiliations:** ^1^School of Economics and Management, Zhejiang University of Science and Technology, Hangzhou, China; ^2^College of Media and International Culture, Zhejiang University, Hangzhou, China

**Keywords:** new media, Internet celebrity economy, entrepreneurial intention, entrepreneurial behavior, questionnaire

## Abstract

Driven by the development of new media, the Internet celebrity economic marketing model has gradually become one of the mainstream online marketing models. It has aroused warm attention on the network platform and provided a breakthrough for entrepreneurship for college students. This thesis aims to explore the influence of the Internet celebrity economy on college students’ entrepreneurial values and entrepreneurial behavior. A questionnaire is conducted among students in two colleges in Xi’an. Moreover, a theoretical model is constructed according to the influence principle of entrepreneurial values on entrepreneurial behavior. The reliability and validity of the questionnaire data are analyzed, and the mediating and moderating effects are tested. The results of the questionnaire show that contemporary college students generally pay attention to Internet celebrity mainly through live broadcast platforms and shopping platforms, among which entertainment and shopping account for the largest proportion. More than 40% of college students are optimistic about the impact of Internet celebrity economy and remain rational and objective on the whole. The results of model analysis show that the standardized path coefficient of entrepreneurial values on entrepreneurial behavior reaches a significant level of 0.85, and entrepreneurial values have a positive and significant impact on entrepreneurial behavior. The influence coefficient of the Internet celebrity economy on entrepreneurial intention is 0.79, and the influence coefficient of entrepreneurial intention on entrepreneurial behavior is 0.84, both reaching a significant level. The entrepreneurial intention has an incomplete intermediary effect in the influence mechanism of the Internet celebrity economy on entrepreneurial behavior. The chain double intermediary composed of entrepreneurial motivation and entrepreneurial intention has an incomplete intermediary effect in the indirect impact path of the Internet celebrity economy on entrepreneurial behavior. The influence coefficient of the product of entrepreneurial intention and entrepreneurial policy satisfaction on entrepreneurial behavior is 0.17, which is always greater than −12.28, indicating that entrepreneurial policy satisfaction has a regulatory effect in the impact path of entrepreneurial intention on entrepreneurial behavior. The research results can guide college students to view the Internet celebrity economy rationally and objectively, and provide some guidance for them to have correct entrepreneurial values.

## Introduction

Entrepreneurship is taken as an essential factor in responding to the challenges of globalization, expanding economic growth and maintaining competitiveness (Urbano et al., 2020). Entrepreneurial values are personal thinking orientation based on the realization, understanding, judgment and choice of entrepreneurship. It is the prerequisite for the sustainable development of entrepreneurial behavior and the basis for college students to choose the correct entrepreneurial value orientation and use effective entrepreneurial methods (Xia and Liu, 2021). College students’ entrepreneurial value orientation has diversified characteristics. They realize their self-worth and help others while pursuing economic interests. From a financial perspective, entrepreneurial values are established based on certain economic factors, and economic indicator is one of the primary standards for shaping entrepreneurial values (Frank et al., 2021). With China’s economy entering the transition period in the new era and the increasing employment pressure, independent entrepreneurship has gradually become a preferred employment mode for college students. The current subversive innovation of social Internet thinking and low-cost business model, and the “Internet +” integration of the Internet and traditional industries provide a wide free network platform for college students’ independent entrepreneurship (Radu et al., 2021; Wu, 2021). Among them, the incubator network is generated based on the growth needs of entrepreneurial teams. It is an innovation network built around the incubation process, an open system of entrepreneurial innovation and an integrated platform for entrepreneurial integration (Wu et al., 2021). According to the analysis on the employment of college students in 2021 of Zhaopin, the graduates who chose freelance employment increased by 8.1% year-on-year compared with 2020, and the graduates who chose independent entrepreneurship increased by 1% year-on-year compared with 2020, accounting for 17.7% of the total number of graduates. College students are adjusting their plans, and more and more college students choose diversified forms of employment to broaden their choices.

In the new media era, Internet celebrities use their special influence and characteristics of the times to revolutionize commodity promotion. Such phenomenon carries more economic value, has a significant social impact, and gradually develops into an Internet celebrity economic business model with the trend of “making fast money” (Al-Emadi and Yahia, 2020; Geng et al., 2020). As a communication channel, the business model mediates between value creation and technology development (Wu W. et al., 2020). However, the Internet celebrity economy is not a short-term outlet, and selling goods through live streaming is gradually normalized. The Internet celebrity economy is gradually rising among college students and showing an increasingly strong development trend, positively or negatively impacting college students’ outlook on job selection (Ahmad, 2020; Wu Y. et al., 2020). Its industry competition is the strongest negative predictor of emotional contagion and cognitive empathy. Moreover, it is also the strongest positive predictor of emotional contagion and cognitive empathy (Sakir et al., 2021). In the *Analysis and Reflection on the Impact of the Webcast and Network Anchor Popularity on Young Students*, it is discussed that the ideas of excessive consumption and hedonic trend output by network anchor affect the construction of college students’ values. Some college students take virtual space as their spiritual support. The present development of the Internet celebrity economy still needs the constraints of relevant national laws and regulations. After considering their own entrepreneurial ability and in-depth understanding of the Internet celebrity economy, college students should establish a correct outlook on employment and career selection, and face up to the temptation of the Internet celebrity economy, which is of great significance to grasp the direction of personal development. Internet celebrity e-commerce economy has the characteristics of novel business model, rich and diverse marketing channels, accurate marketing positioning, strong interaction with buyers, and low cost but strong liquidity. It is in line with modern “short, flat and fast” consumption model (Guo, 2022). Moreover, the maturity of Internet information technology, the development of we media and financial media, and the rapid progress of e-commerce and live broadcasting platforms provide new opportunities for innovation and entrepreneurship for college students (Che et al., 2022). “The same network and speed” in rural and urban areas has opened up the market and escorted students’ innovation and entrepreneurship (Lin and Cheung, 2022). The online payment market is full of the resilience and provides a new path for students to innovate and start businesses (Ngai, 2022). The scale of e-commerce live broadcast users has increased sharply, providing a new fulcrum for students’ innovation and entrepreneurship (Sandel and Wang, 2022). This exploration will study college students’ entrepreneurship under the influence of the Internet celebrity economy. By analyzing college students’ entrepreneurial values and entrepreneurial behavior, some suggestions conducive to college students’ entrepreneurial ability under the Internet celebrity economy are put forward to develop the pattern of college students’ innovation and entrepreneurship.

The existing research shows that the Internet celebrity economy has a certain impact on the construction of college students’ values, but there is no in-depth study on the impact mechanism. With the increasingly severe employment situation, college students generally encounter obstacles in their actual employment. Due to the low cost and high income of the Internet celebrity economy, college students’ entrepreneurial values and entrepreneurial behavior will be affected to a certain extent. Based on this, first, the questionnaire is designed to explore college students’ cognition and views on the Internet celebrity and Internet celebrity economy. Besides, the theoretical model of the influence mechanism of college students’ entrepreneurial values on entrepreneurial behavior under the environment of the Internet celebrity economy has been creatively constructed. Moreover, relevant research hypotheses are put forward. Finally, statistical software is used to analyze the questionnaire results and draw the following conclusions. Entrepreneurial values have a direct positive and significant impact on entrepreneurial behavior, and entrepreneurial intention has an incomplete intermediary effect in the impact mechanism of the Internet celebrity economy on entrepreneurial behavior. The chain double intermediary composed of entrepreneurial motivation and entrepreneurial intention has an incomplete intermediary effect in the indirect impact path of online Red economy on entrepreneurial behavior. Entrepreneurial policy satisfaction has a moderating effect in the impact path of entrepreneurial intention on entrepreneurial behavior. The purpose is to provide some reference for exploring the influence mechanism of the Internet celebrity economy on college students’ entrepreneurial values and entrepreneurial behavior.

## Research Status

At present, there is no unified and systematic explanation for the definition of “Internet celebrity.” According to the interpretation of the Chinese dictionary, “Internet celebrity” refers to people who gradually become popular because an event or behavior is concerned by netizens in real or online life. Some scholars believe that “Internet celebrity” is a specific person. Duan et al. (2022) believed that “Internet celebrity” is a social phenomenon (Duan et al., 2022); Gabinete et al. (2022) thought that “Internet celebrity” is a domestic social phenomenon, which limits its regional scope (Gabinete et al., 2022). Most scholars believe that the development of “Internet celebrity” phenomenon has three stages: text age, graphic age, and video age (Jia et al., 2022). However, a few scholars study the “Internet celebrity” in different periods (Joshi et al., 2022). The main research directions of the Internet celebrity economy include the connotation of the Internet celebrity economy, the problems existing in the development of the Internet celebrity economy, the consumption behavior of the Internet celebrity economy, and the development prospect of the Internet celebrity economy. Kinal (2022) believed that the Internet celebrity economy was a social phenomenon, a new economic model and the composition of fans’ transformed purchasing power (Kinal, 2022). The business of the Internet celebrity mainly depends on the Internet celebrity itself. As an intermediary of communication, Internet celebrity links consumers and products to maximize the benefits of marketing. Lv et al. (2022) pointed out that multiple problems existed in the development of the Internet celebrity economy, such as the Internet celebrity’s short life cycle, the lack of trust of consumers, the serious content homogenization and vulgarization, and the lack of a relatively perfect system (Lv et al., 2022); Özmen et al. (2022) held that the Internet celebrity economy greatly impacted the consumption behavior of young people and establishing a correct and healthy consumption concept and preventing an impetuous atmosphere were necessary (Özmen et al., 2022); Tridalestari et al. (2022) pointed out that as a new economic force, the Internet celebrity economy had many problems in its development, but its development prospect was unstoppable (Tridalestari et al., 2022).

There are few studies on the combination of the Internet celebrity economy and entrepreneurship. The Internet celebrity economy belongs to the field of e-commerce. Therefore, the entrepreneurship research of college students in the field of e-commerce is analyzed here. In the current Chinese economic environment, the continuous development of Internet information technology and related business models promotes the rapid arrival of China’s Internet and affects all aspects of people’s life. In this context, the network infrastructure is continuously improved, and the innovation and creativity of society are rapidly improved. It has led to continuous changes and progress in China’s overall economic structure, especially innovation-driven development, which has continuously enhanced the domestic economic structure‘s Internet trend and achievements (Wu and Chen, 2021); Wu et al. (2021) pointed out that under the Internet action plan, modern Internet technologies, such as cloud storage, big data technology and the Internet of things, could effectively promote the innovative development of traditional industries and the continuous progress of emerging industries (Wu et al., 2021). With the progress of the Internet industry, the demand for Internet talents is increasing, and the overall number of social graduates has increased significantly. Many scholars have done a lot of research on college students’ innovation and entrepreneurship under the Internet environment. Wu et al. (2022) pointed out in the research and analysis of the direction of college students’ innovation and entrepreneurship that the Internet provided college students with many entrepreneurial choices, but for different college students, the appropriate choice was an important factor for the success of entrepreneurship. In the current Internet entrepreneurship environment, e-commerce, scientific and technological products, intellectual services and chain franchises are the main directions of college students’ entrepreneurship (Wu et al., 2022). Moreover, it is believed that college students’ entrepreneurship should be well planned according to their professional ability, skill level, team and capital. According to the investigation of college students’ innovation and entrepreneurship under the Internet environment, Yazdani et al. (2022) found that college students’ innovation and entrepreneurship still faced a series of problems. These problems are mainly in the lack of social experience and entrepreneurship education and training, the acquisition of entrepreneurship funds and the difficulty of obtaining preferential policies for entrepreneurship (Yazdani et al., 2022); Yu and Zhao (2022) believed that college students’ entrepreneurship in the Internet environment should receive more support. Especially in the context of mass entrepreneurship and innovation, more entrepreneurship education and training and entrepreneurship policy preferences should be given to guide the development and implementation of college students’ entrepreneurship projects (Yu and Zhao, 2022).

The Internet celebrity economy is a social phenomenon. There is less research on its combination with college students’ entrepreneurship. Therefore, combining the two here is conducive to filling the blank of research in this field. Zhang (2022) found that the Internet celebrity economy impacts the values of college students (Zhang, 2022). Therefore, combined with previous studies, this exploration studies the entrepreneurial behavior and entrepreneurial values of college students under the Internet celebrity economy, and tries to get suggestions that can improve the entrepreneurial rate of college students.

## Research on the Influence of Internet Celebrity Economy on College Students’ Entrepreneurial Values and Entrepreneurial Behavior

**FIGURE 1:**
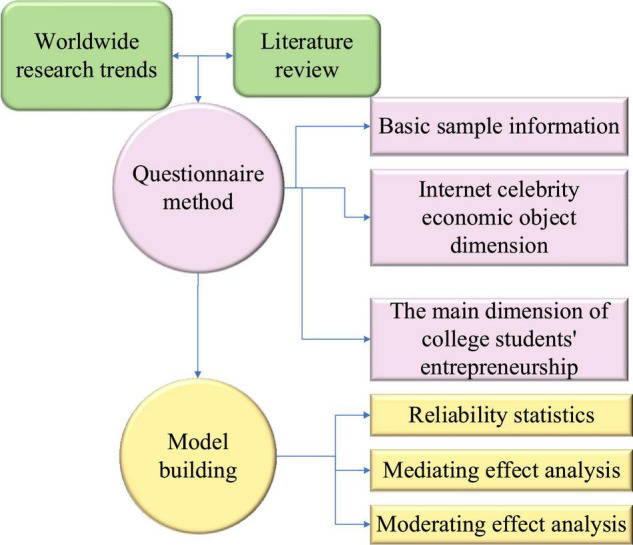
Research methods.

College students are the reserve force of human resources for social development. In the trend of innovation and entrepreneurship and the Internet celebrity economy, diversified entrepreneurial ideas always affect their entrepreneurial values and entrepreneurial behavior. Figure 1 shows the overall research methods.

### Questionnaire

By consulting relevant literature, the questionnaire was designed according to the basic principles of sociology and statistics (Rogoza et al., 2018). 28 related questions were set. The five-point method was adopted, and one of the five options was selected. They were “very inconsistent/agreed,” “relatively inconsistent/agreed,” “uncertain,” “relatively consistent/agreed” and “very consistent/agreed” (Elena et al., 2021; Marquardt et al., 2021). The Supplementary Appendix displays the specific questionnaire.

The survey samples were students (including master and doctoral students) from Xi’an Jiaotong University and Xidan University. A random sampling survey was conducted. Questionnaire Star applet was used to release the questionnaire and sort and analyze the background data to ensure authenticity and reliability. After three days, 262 questionnaires were collected and 246 were valid, with an effective rate of 93.89%.

### Theoretical Model Construction of the Influence Mechanism of Entrepreneurial Values on Entrepreneurial Behavior

**FIGURE 2:**
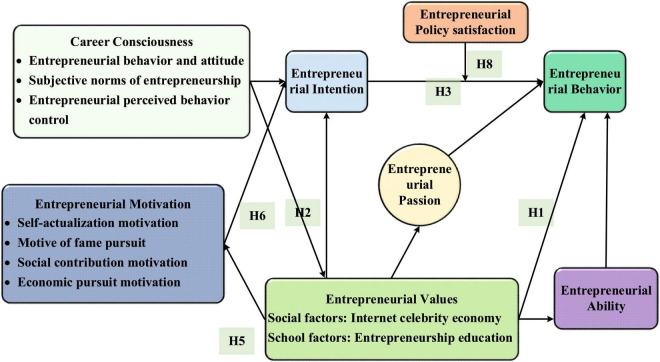
Theoretical model of the influence mechanism of college students’ entrepreneurial values on entrepreneurial behavior.

Based on the planned behavior theory, self-efficacy theory and triadic reciprocal determinism, a theoretical model of the impact mechanism of college students’ entrepreneurial values on entrepreneurial behavior in the Internet celebrity economy environment is constructed (Skaalvik, 2020; Black et al., 2021; Zheng et al., 2021; Figure 2).

H1-H8 (except for the mediating effect hypotheses H4 and H7) are the hypotheses of helping influence among components, and the mediating effect influence is not shown in the model. In the model, entrepreneurial values refer to the ideological basis for college students to determine correct entrepreneurial goals and adopt effective entrepreneurial methods, and the judgment and selection criteria for entrepreneurial behavior (Piras et al., 2021). The shaping of entrepreneurial values has two dimensions: social factors and school factors. Social factors include the impact of the Internet celebrity economy, and school factors include the development of entrepreneurship education. Entrepreneurship education will involve individual uniqueness and subjectivity. It is necessary to consider individual differences and special education needs and build a flexible education model (Yuan and Wu, 2020). The following hypotheses are put forward based on the model and the review:

(1) Research hypotheses of the direct impact mechanism of Internet celebrity economy on entrepreneurial behavior.
**H1:** entrepreneurial values directly and significantly affect entrepreneurial behavior.
**H1-1:** entrepreneurial values directly and significantly affect entrepreneurial behavior under the influence of the Internet celebrity economy.
**H1-2:** entrepreneurial values directly and significantly affect entrepreneurial behavior under the influence of entrepreneurship education.
(2) Research hypotheses of the influence mechanism of Internet celebrity economy on entrepreneurial behavior with entrepreneurial intention as a single mediating.

**FIGURE 3:**
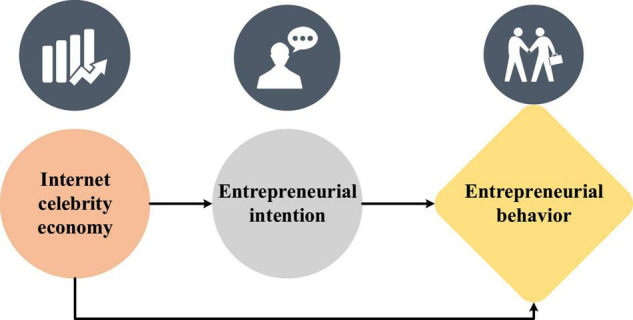
Relationship among variables of Internet celebrity economy, entrepreneurial intention and entrepreneurial behavior.

Entrepreneurial intention refers to the mentality of guiding individuals’ attention and experience toward planned entrepreneurial behavior (Wu et al., 2019). Figure 3 shows the relationship among the three variables of Internet celebrity economy, entrepreneurial intention and entrepreneurial behavior.

(1) Internet celebrity economy and entrepreneurial intention.
**H2:** the Internet celebrity economy has a significant positive impact on entrepreneurial intention.
(2) Entrepreneurial intention and entrepreneurial behavior.
**H3:** entrepreneurial intention has a significant positive impact on entrepreneurial behavior.
(3) The mediating effect of entrepreneurial intention and Internet celebrity economy on entrepreneurial behavior.
**H4:** entrepreneurial intention has a mediating effect in the impact mechanism of Internet celebrity economy on entrepreneurial behavior.
(4) Research hypotheses on the influence mechanism of the Internet celebrity’s chain double mediating composed of entrepreneurial intention and entrepreneurial motivation on entrepreneurial behavior.

**FIGURE 4:**
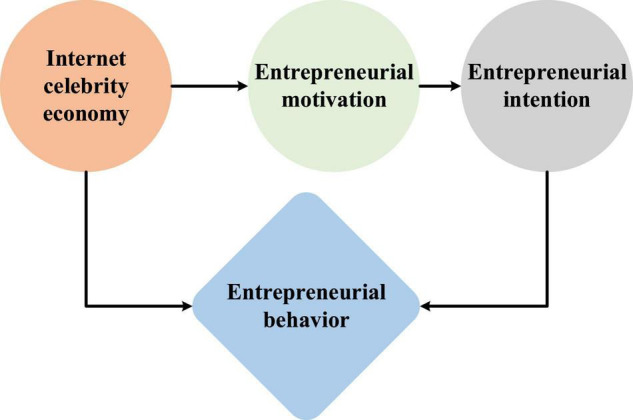
Relationship among variables of the Internet celebrity economy, entrepreneurial intention, entrepreneurial motivation and entrepreneurial behavior.

A critical active feedback-seeking behavior is usually interpreted as an antecedent-oriented behavior based on three primary motives (Qian et al., 2018). Figure 4 shows the influence relationship among the four variables of the Internet celebrity economy, entrepreneurial intention, entrepreneurial motivation and entrepreneurial behavior.

(1) Internet celebrity economy and entrepreneurial motivation.

Entrepreneurial motivation is divided into four dimensions: self-realization, reputation pursuit, social contribution and economic pursuit. The following hypotheses are made.

**H5:** the Internet celebrity economy has a significant positive impact on entrepreneurial motivation.
**H5-1:** the Internet celebrity economy has a direct and significant positive impact on self-realization motivation.
**H5-2:** the Internet celebrity economy has a direct and significant positive impact on reputation pursuit motivation.
**H5-3:** the Internet celebrity economy has a direct and significant positive impact on social contribution motivation.
**H5-4:** the Internet celebrity economy has a direct and significant positive impact on the motivation of economic pursuit.

(2) Entrepreneurial intention and entrepreneurial motivation.
**H6:** entrepreneurial motivation has a significant positive impact on entrepreneurial intention.
**H6-1:** self-realization motivation has a significant positive impact on entrepreneurial intention.
**H6-2:** reputation pursuit motivation has a significant positive impact on entrepreneurial intention.
**H6-3:** social contribution motivation has a significant positive impact on entrepreneurial intention.
**H6-4:** economic pursuit motivation has a significant positive impact on entrepreneurial intention.

(3) There is a mediating effect in the chain double mediating composed of entrepreneurial motivation and entrepreneurial intention.
**H7:** the chain double mediating composed of entrepreneurial motivation and entrepreneurial intention has a mediating effect in the indirect impact of the Internet celebrity economy on entrepreneurial behavior.
(4) Research hypotheses on the moderating effect of entrepreneurial policy satisfaction on the impact mechanism of entrepreneurial intention on entrepreneurial behavior based on the impact of the Internet celebrity economy.

**FIGURE 5:**
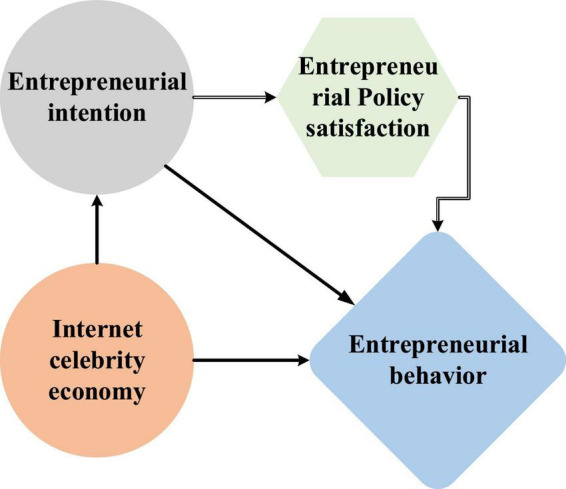
The influence relationship among the four variables of the Internet celebrity economy, entrepreneurial intention, entrepreneurial policy satisfaction and entrepreneurial behavior.

Figure 5 shows the influence relationship among four variables: the Internet celebrity economy, entrepreneurial intention, entrepreneurial policy satisfaction and entrepreneurial behavior.

Entrepreneurial policy satisfaction is introduced into the model as a moderator variable to explore its moderating effect. Moderator variables generally construct the product term of moderator variables and independent variables, and then use the multi-level linear model/regression equation or structural equation to test the interaction. In actual entrepreneurship, if individuals have higher satisfaction with entrepreneurship policies, it will enhance individual entrepreneurial intention. The following hypothesis is made:

**H8:** entrepreneurial policy satisfaction has a moderating effect in the impact mechanism of entrepreneurial intention on entrepreneurial behavior.

### Model Verification Method

#### Source Data Processing and Scale Verification

The original data of this model is questionnaire survey data. The effective questionnaire is sorted, coded, entered into Statistical Product and Service Solutions (SPSS) software, and archived. When processing data, each option is coded as 1-5 in turn, and SPSS and Analysis of Moment Structure (AMOS) software are used for data analysis. The designed data analysis methods include reliability and validity analysis, path analysis, mediating effect test, and regulatory effect test (Vielma-Aguilera et al., 2021; Yee, 2021). SPSS26.0 software is used to test the reliability of Cronbach’s alpha coefficient of each scale. AMOS26.0 software is adopted to test the scale’s validity through confirmatory factor analysis, ensure the reliability and effectiveness of the scale, and lay the foundation for subsequent tests and analysis. AMOS is a powerful structural equation modeling software, so the influence path coefficients between variables are also obtained through the software analysis. The statistical work can be carried out on any computer with the two software installed.

#### Mediating Effect Test

**FIGURE 6:**
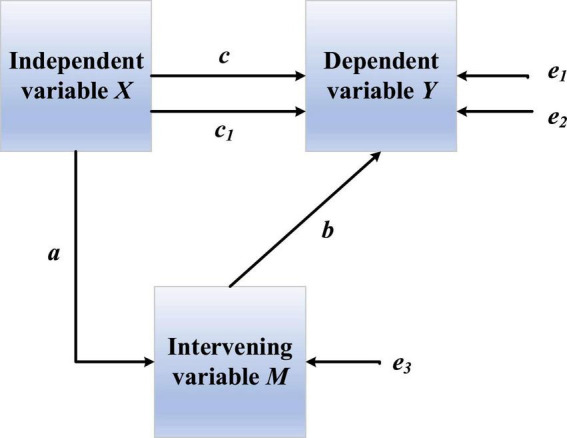
Relationship diagram of variables in mediating effect.

Mediating effect models are widely used in the research fields of psychology and social sciences to analyze the influence process and mechanism of independent variables on dependent variables (Arslan, 2021). When variable 1 affects variable 2 through intermediate variables, intermediate variables are mediators to explain the internal mechanism of the relationship. Figure 6 shows the relationship of variables.

There is a certain relationship among variables, which can be calculated. This model tests the mediating effect of hypotheses 2–7 through the stepwise test regression coefficient method (Chen, 2019). The stepwise test regression equation is:


(1) $$Y=c\,X+e_{1}$$

(2) $$M=a\,X+e_{2}$$

(3) $$Y=c_{1}\,X+b\,M+e_{3}$$

where *X* is the independent variable, *Y* is the dependent variable and *M* is the mediator. Coefficients *a*, *b* and *c* are effect values.

The specific steps are:

(1) Calculating the total effect *c* of *X* and *Y* in each hypothesis;
(2) Calculating the effect *a* of *X* on the mediator *M* in each hypothesis and the effect *b* of mediator *M* on *Y*;
(3) Controlling the mediator *M* and calculating the direct effect *c*_1_ of *X* on *Y*;

If the total effect *c* is significant and the effects *a* and *b* are significant, the mediating effect is significant. On the contrary, it is not significant. Further, if the effect *c*_1_ is significant, there is partial mediation; if the effect *c*_1_ is not significant, it is complete mediation.

#### Moderating Effect Test

When the relationship between the two variables depends on the third variable, there is a moderating effect. The third variable is the moderator variable. Moderator variables explain the changes of the relationship between the two variables under different conditions. It is generally described that under the condition of moderator variables, variable 1 has a greater impact on variable 2 (Friend et al., 2020; Martín-Alcázar et al., 2020). In the moderating effect test, it is necessary to construct the interaction product, test whether the interaction term is significant by path analysis, and deduce the corresponding moderating effect. In this model, entrepreneurial policy satisfaction is the moderating variable, and the product term of entrepreneurial policy satisfaction of entrepreneurial intention is the interactive term. The structural equation model is used to test the significance of the interactive term and verify hypothesis 8.

## Questionnaire Survey and Analysis of Entrepreneurial Prediction Results

### Descriptive Statistical Analysis of Questionnaire Samples

#### Survey Sample Analysis

The diversity of individual attributes can enrich the interpretation of the research results, and individuals with some attributes may have relatively strong entrepreneurial motivation (Wu and Song, 2019). The survey objects cover a wide range and are representative. Table 1 shows the results of basic information.

**TABLE 1:** Basic information of survey samples.

Types	Variable	Quantity	Proportion
Gender	Male	113	45.93%
	Female	133	54.06%
Political affiliation	CPC member (including preparatory)	25	10.16%
	Member of the Chinese Communist Youth League	208	84.55%
	General public	6	2.44%
	Others	7	2.85%
Grades	Freshman	41	16.67%
	Sophomore	39	15.85%
	Junior	62	25.21%
	Senior	76	30.89%
	Senior 5 (medical)	8	3.25%
	Master and doctoral students	20	8.13%
Major	Science and engineering	61	24.79%
	Literature	54	21.95%
	Agriculture and forestry	35	14.22%
	Medical science	25	10.17%
	Arts	31	12.61%
	Other majors	40	16.26%

#### College Students’ Cognition of Internet Celebrity Economy

**FIGURE 7:**
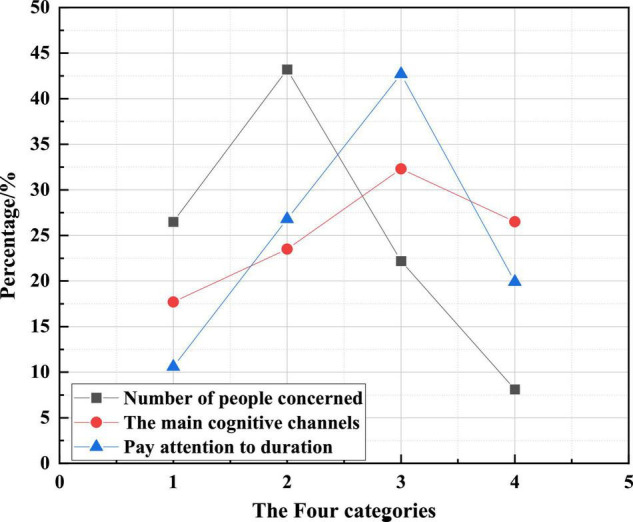
Basic situations of college students’ attention to Internet celebrity.

Figure 7 shows the basic situation of college students’ attention to Internet celebrities.

Abscissa 1, 2, 3, and 4 represent different meanings in the three curves. In the curve of the number of people concerned, they are special attention, general attention, occasional attention and no attention. In the curve of the main understanding channel, they are live broadcast platform, video software, shopping website and social software. In the curve of average attention time, they are less than 1 h, 1–2 h, 2–3 h and more than 3 h.

Figure 7 shows that 91.9% of college students pay attention to Internet celebrities to varying degrees, and college students are one of the main audience groups of Internet celebrities. The highest proportion of college students who often pay attention to Internet celebrities is 43.2%. The main channels for them to know Internet celebrities are live broadcasting and shopping platforms. More than 42% of college students spend 2–3 h browsing Internet celebrities-related information every day, and only 10.6% of college students spend less than 1 h on average.

**FIGURE 8:**
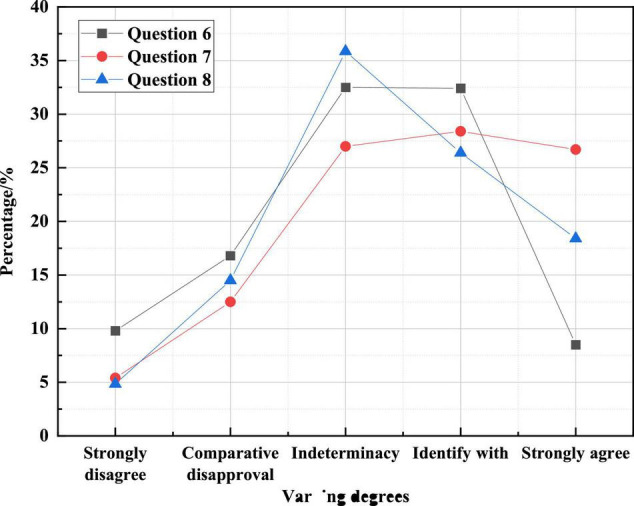
Distribution map of college students’ views on Internet celebrity economy-related cases in the questionnaire.

Figure 8 shows college students’ views on the relevant examples of Internet celebrity economy in the questionnaire.

Figure 8 shows that more than 40% of college students are optimistic about the impact of the Internet celebrity economy in each case, about 30% of college students still maintain a rational wait-and-see attitude, and few college students disagree with the Internet celebrity economy. The key to surviving in a highly dynamic market is to maintain a long-term competitive advantage, and new product development is the fulcrum of competitive organizational strategy. The Internet celebrity economy is considered to have a high hype component and is not a stable, sustainable and healthy economic model.

### Analysis of the Theoretical Model of the Impact of Entrepreneurial Values on Entrepreneurial Behavior

#### Reliability and Validity Test Results of Entrepreneurial Values Scale and Entrepreneurial Intention

**FIGURE 9:**
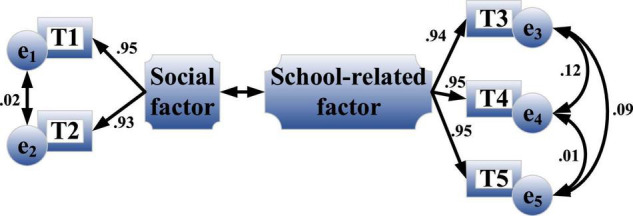
Results of confirmatory factor analysis of entrepreneurial values scale.

The entrepreneurial values scale is measured in two dimensions. Figure 9 is the confirmatory factor analysis results of the entrepreneurial values scale obtained by parameter estimation with the maximum likelihood estimation method.

The rectangular variables in the figure are observation variables, which specifically represent the scale items, such as T1 and T2. Circular variables represent error terms, such as e_1_ and e_2_. The reliability test results of the scale show that the reliability of social factors is 0.951, the reliability of school factors is 0.983, and the overall reliability is 0.983. It proves that the entrepreneurial values scale used is highly reliable.

**FIGURE 10:**
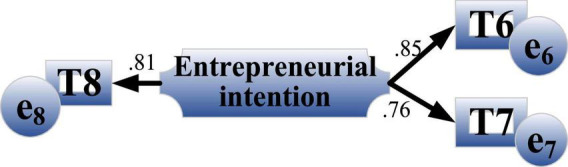
Confirmatory factor analysis results of entrepreneurial intention.

Three items are used to measure entrepreneurial intention: the possibility of starting a business from the media during school, the possibility of freelancing within three years after graduation and the willingness to take risks. Figure 10 shows the results of confirmatory factor analysis of entrepreneurial intention.

The reliability of the entrepreneurial intention scale is 0.86, which is high. The factor load values measured by the three items of the entrepreneurial intention scale are 0.85, 0.76 and 0.81, respectively, which can better reflect the dimension information.

#### Analysis of the Direct Impact of Entrepreneurial Values on Entrepreneurial Behavior

**FIGURE 11:**
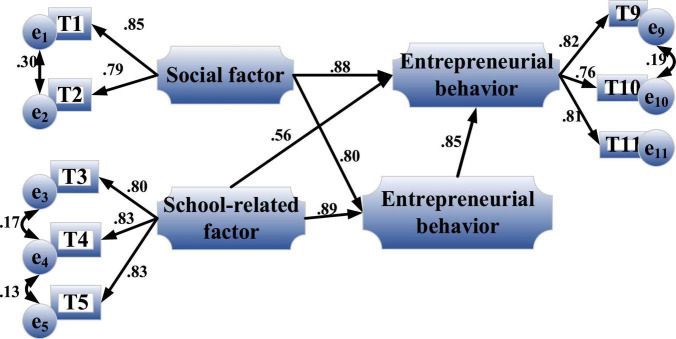
Path coefficient diagram of the direct impact of entrepreneurial values on entrepreneurial behavior.

Figure 11 is the path coefficient diagram of the direct impact of entrepreneurial values on entrepreneurial behavior after minor residual correction.

Figure 11 shows that the standardized path coefficient of entrepreneurial values on entrepreneurial behavior reaches a significant level, 0.85, *P* < 0.001, indicating that entrepreneurial values have a positive and significant impact on entrepreneurial behavior, and H1 is established. The influence coefficient of social and school factors on entrepreneurial behavior is 0.88, reaching a significant level. H1-1 is established, and there is a direct positive significant impact. Similarly, H1-2 does not hold.

#### An Analysis of the Single Mediating Effect

**FIGURE 12:**
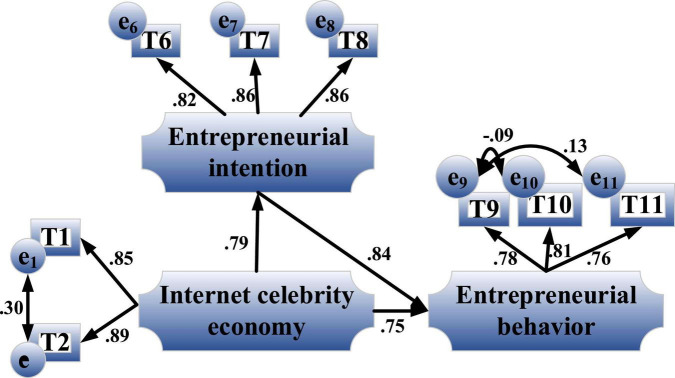
Path coefficient diagram of the indirect impact of the Internet celebrity economy with entrepreneurial intention as a single mediating on entrepreneurial behavior.

Figure 12 is the path coefficient diagram of the indirect impact of the Internet celebrity economy with entrepreneurial intention as a single mediating on entrepreneurial behavior.

Figure 12 shows that the influence coefficient of Internet celebrity economy on entrepreneurial intention is 0.79, *P* < 0.05, reaching a significant level, and the influence coefficient of entrepreneurial intention on entrepreneurial behavior is 0.84, *P* < 0.05. Meanwhile, the total effect is 0.75, which is significant. Hence, the mediating effect is significant, H2-4 are established, and there is an incomplete mediating.

#### Analysis of Chain Double Mediating Effect

**FIGURE 13:**
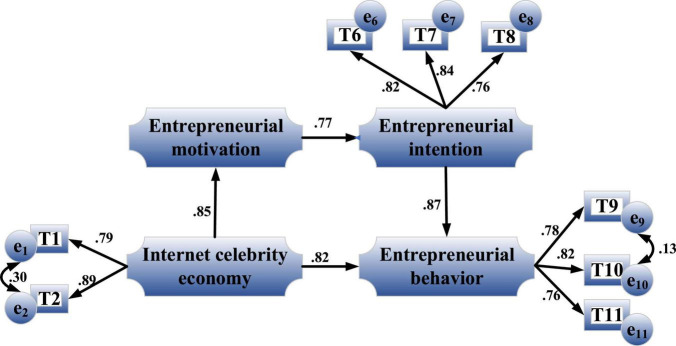
Path coefficient diagram of the indirect impact of the Internet celebrity economy with entrepreneurial motivation and entrepreneurial intention as the chain double mediating on entrepreneurial behavior.

Figure 13 is the path coefficient diagram of the indirect impact of the Internet celebrity economy with entrepreneurial motivation and entrepreneurial intention as the chain double mediating on entrepreneurial behavior.

**FIGURE 14:**
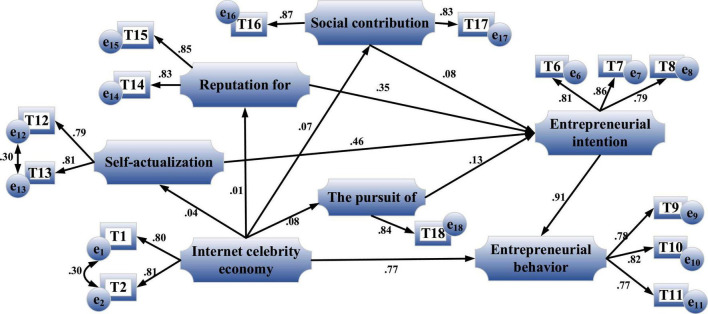
Path coefficient diagram of the indirect impact of the Internet celebrity economy with the modified chain double mediating on entrepreneurial behavior.

Figure 14 is the path coefficient diagram of the indirect impact of the Internet celebrity economy with the modified chain double mediating on entrepreneurial behavior.

The influence coefficient of the Internet celebrity economy on entrepreneurial motivation is 0.85, reaching a significant level, *P* < 0.05. Hence, H5 is established. Next, the influence coefficient of entrepreneurial motivation on entrepreneurial intention is 0.77, reaching a significant level, *P* < 0.01. Therefore, H6 is established. Besides, the influence coefficient of entrepreneurial intention on entrepreneurial behavior is 0.87, reaching a significant level, and the influence coefficient of the Internet celebrity economy on entrepreneurial behavior is 0.82, reaching a significant level. Therefore, entrepreneurial motivation and entrepreneurial intention play a mediating role in the impact mechanism of the Internet celebrity economy on entrepreneurial behavior. The mediating effect is 0.57, which is an incomplete mediating. Therefore, H7 is established. Similarly, in Figure 14, the influence coefficients in H5-1, H5-2, H5-3, and H5-4 are not significant, and the hypotheses do not hold.

#### Entrepreneurial Policy Satisfaction

**FIGURE 15:**
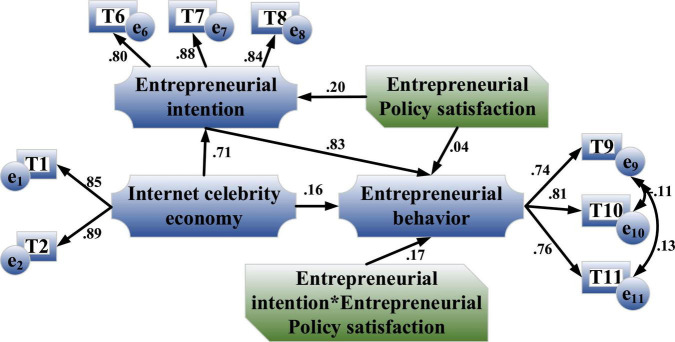
Path coefficient diagram of the moderating effect of entrepreneurial policy satisfaction in the impact mechanism of entrepreneurial intention on entrepreneurial behavior.

Figure 15 is the path coefficient diagram of the moderating effect of entrepreneurial policy satisfaction in the impact mechanism of entrepreneurial intention on entrepreneurial behavior.

Figure 15 shows that the influence coefficient of the product term of entrepreneurial intention and entrepreneurial policy satisfaction on entrepreneurial behavior is 0.17, which is always greater than −12.28. It indicates that entrepreneurial policy satisfaction can play a significant moderating effect in entrepreneurial intention and entrepreneurial behavior, and H8 is established.

## Conclusion

The development of the Internet celebrity economy under the background of Internet commerce has had a certain impact on contemporary college students’ entrepreneurial values and entrepreneurial behavior. However, there is less research on the internal correlation mechanism of specific impact. First, the questionnaire is designed to explore college students’ cognition and views on Internet celebrities and the Internet celebrity economy. Next, the theoretical model of the influence mechanism of college students’ entrepreneurial values on entrepreneurial behavior under the environment of the Internet celebrity economy is creatively constructed, and the relevant research hypotheses are put forward. Finally, SPSS and AMOS statistical software are used to analyze the reliability and validity of the scale and the survey results. The questionnaire results show that contemporary college students generally pay attention to Internet celebrities, among which the purpose of entertainment and shopping accounts for the largest proportion. More than 40% of college students hold a rational and optimistic attitude toward the Internet celebrity economy. The model analysis results show that entrepreneurial values have a direct positive and significant impact on entrepreneurial behavior. The entrepreneurial intention has an incomplete mediating effect in the influence mechanism of the Internet celebrity economy on entrepreneurial behavior. The chain double mediating composed of entrepreneurial motivation and entrepreneurial intention has an incomplete mediating effect in the indirect impact path of Internet celebrity economy on entrepreneurial behavior. Entrepreneurial policy satisfaction has a moderating effect on the impact path of entrepreneurial intention on entrepreneurial behavior. Due to the limited time, only hypotheses on two influencing factors are put forward, and multiple factors can be explored in the future, such as whether the Internet celebrity economy has an impact on students’ entrepreneurial policy satisfaction, and whether the entrepreneurial policy satisfaction plays an intermediary role in the impact mechanism of the Internet celebrity economy on entrepreneurial behavior. The research results can guide college students to view the Internet celebrity economy rationally and objectively, and provide some guidance for them to establish correct entrepreneurial values. The theoretical contribution is to clarify the relationship between entrepreneurial values and entrepreneurial behavior, which lays a theoretical foundation for later research in the same field. The practical research value is that after further improving the research, the research results can be applied to college students’ entrepreneurship under the network economy to improve the rate of their entrepreneurship and success.

## Data Availability Statement

The raw data supporting the conclusions of this article will be made available by the authors, without undue reservation.

## Ethics Statement

The studies involving human participants were reviewed and approved by Zhejiang University of Science and Technology Ethics Committee. The patients/participants provided their written informed consent to participate in this study. Written informed consent was obtained from the individual(s) for the publication of any potentially identifiable images or data included in this article.

## Author Contributions

Both authors listed have made a substantial, direct, and intellectual contribution to the work, and approved it for publication.

## Conflict of Interest

The authors declare that the research was conducted in the absence of any commercial or financial relationships that could be construed as a potential conflict of interest.

## Publisher’s Note

All claims expressed in this article are solely those of the authors and do not necessarily represent those of their affiliated organizations, or those of the publisher, the editors and the reviewers. Any product that may be evaluated in this article, or claim that may be made by its manufacturer, is not guaranteed or endorsed by the publisher.
