# Supplementary material for: The Correlation Analysis for New Media Internet Celebrity Economy in College Students’ Entrepreneurial Values and Entrepreneurial Behavior
Source: Front Psychol. 2022 Jun 16;13:892347. doi: 10.3389/fpsyg.2022.892347 (PMC9244789; doi:10.3389/fpsyg.2022.892347)
Supplement: Supplementary file 1 [file Data_Sheet_1.docx]

**Appendix**

Questionnaire on entrepreneurial concept and tendency of contemporary college students

Dear students,

Hello! We are the entrepreneurship research group of XXX college in XXX school. This investigation is conducted to understand the impact of the Internet celebrity economy on contemporary college students' entrepreneurial values and entrepreneurial behavior under new media backgrounds. The questionnaire is anonymous, and the background uniformly processes the data. Please choose according to your real ideas. This survey is only for subject research, not for commercial use. Thank you for participating and providing your views and suggestions. It is our great honor. Thank you!

Part 1:basic information

1. Your gender: ()

AMale BFemale

2. Your political affiliation: ()

A CPC member (including preparatory)

B Member of the Chinese Communist Youth League

C General public

D Other

3. Your grade:()

A Freshman

B Sophomore

C Junior

D Senior

E Senior 5 (medical)

F Master and doctoral students

4. Your major ()

A Science and engineering

B Literature
C Agriculture and forestry

D Medical science
E Arts
F Other majors

5. Have you paid attention to "Internet celebrity"? ( )

A Frequent attention

BGeneral attention

COccasional attention

DNo attention

6. Your main attention channels for "Internet celebrity". ( ) Multiple choices are allowed

ATiktok, bilibili and other video software

B Microblog, Wechat and other social software

CLive broadcast platform software such as DouYu and Huya

D Taobao, Jingdong and other shopping websites

EOther

7. In 2016, the advertising of papi sold 22 million. What do you think of this phenomenon? ( )

A It's worth, and the content is innovative.

B Understand. This is the embodiment of the value of "Internet celebrity".

C Don't understand. This is the excessive flaunting of the value of "Internet celebrity".

DIt doesn't matter. Having no idea

E Other

8. In the general list of live e-commerce anchor, Gross Merchandise Volume (GMV), in the first quarter of 2021, Weiya contributed RMB 7.15 billion, Li Jiaqi contributed RMB 4.5 billion, and Simba contributed RMB 2.15 billion. The total GMV of the three reached RMB 13.8 billion, accounting for 37.3% of the total turnover of GMV TOP50 in the first quarter. What do you think of this phenomenon? ( )

A It is worth encouraging. It can promote economic development

B Understand. There are often discounts in the anchor room.

C Don't understand. It is easy for consumers to immerse themselves in the pleasure of consumption and exceed their purchasing power

DIt doesn't matter. Having no idea

E Other

9. On October 17, 2021, a technology video blogger named “@ Hi, teacher! I’m He” released a new video. The leg of the landing table used in the video was LIFT1 of Loctek. The second day after the video was released, at the opening on Monday, October 8, the shares of sponsor Loctek of the video rose sharply. After the closing, Loctek's shares were 25.20 yuan / share, up 13.51%, and the market value increased by nearly 590 million yuan. What do you think of this phenomenon? ( )

A Support. It has good publicity and promotion effect on business promotion.

B Understand. Videos attract traffic for goods and create gimmicks.

C Don't understand. Fans blindly follow the trend of consumption.

DIt doesn't matter. Having no idea

E Other

10. What are the main reasons why you pay attention to "Internet celebrity"? () Multiple choices are allowed

A Entertainment

B Conformity psychology

C Consumption options

D Learning information

E Expand social networking

F Part-time jor and entrepreneurship

G Others

Part 2: the dimension of Internet celebrity economic object

| 1 | I understand the Internet celebrity economy. | 1 | 2 | 3 | 4 | 5 |
| --- | --- | --- | --- | --- | --- | --- |
| 2 | I intend to become an Internet celebrity to start a business. | 1 | 2 | 3 | 4 | 5 |
| 3 | I take the initiative to take entrepreneurship education courses to accumulate a theoretical basis. | 1 | 2 | 3 | 4 | 5 |
| 4 | I often take the initiative to participate in various entrepreneurship lectures and skills training. | 1 | 2 | 3 | 4 | 5 |
| 5 | I often take the initiative to participate in entrepreneurial events, such as college students' innovation and entrepreneurship competitions. | 1 | 2 | 3 | 4 | 5 |
| 6 | I am willing to start a business and take risks if I have the opportunity. | 1 | 2 | 3 | 4 | 5 |
| 7 | I have the idea of starting a business when I am in school. | 1 | 2 | 3 | 4 | 5 |
| 8 | I will choose to start my own business as soon as possible after graduation. | 1 | 2 | 3 | 4 | 5 |
| 9 | I am willing to prepare and take action for entrepreneurship. | 1 | 2 | 3 | 4 | 5 |
| 10 | I always pay attention to entrepreneurial opportunities and communicate with teachers. | 1 | 2 | 3 | 4 | 5 |
| 11 | I have started my entrepreneurial activities and are still in progress. | 1 | 2 | 3 | 4 | 5 |
| 12 | Becoming an Internet celebrity can have the opportunity to cooperate with famous experts in various fields. | 1 | 2 | 3 | 4 | 5 |
| 13 | Entrepreneurship can bring a sense of achievement and challenge yourself. | 1 | 2 | 3 | 4 | 5 |
| 14 | Becoming an Internet celebrity can enhance personal popularity and reputation and win respect. | 1 | 2 | 3 | 4 | 5 |
| 15 | Internet celebrities have influence, can participate in various important meetings as representatives, and have the right to speak. | 1 | 2 | 3 | 4 | 5 |
| 16 | Becoming an Internet celebrity can publicize correct values and affect the young generation. | 1 | 2 | 3 | 4 | 5 |
| 17 | Internet celebrity economy can promote social and economic development and improve development efficiency. | 1 | 2 | 3 | 4 | 5 |
| 18 | Becoming an Internet celebrity can realize economic independence and accumulate wealth as soon as possible. | 1 | 2 | 3 | 4 | 5 |
